# Efficacy and Safety of High-Frequency Optical Coherence Tomography (HF-OCT) for Coronary Imaging: A Multicenter Study

**DOI:** 10.1016/j.jscai.2025.102577

**Published:** 2025-03-18

**Authors:** Donald L. Quimby, Eric S. Rothstein, Henry C.T. Richmond, Emmanuel Bassily, Bibhu D. Mohanty, Robert Sawyer, Michael Shih, Michael N. Young, Amit P. Amin, Hannah Chaudry, Jimmy Devries, Michael R. Jones, Fadi Matar, Aaron V. Kaplan, Giovanni J. Ughi, Hiram G. Bezerra

**Affiliations:** a Interventional Cardiology Center, Tampa General Hospital, Tampa, Florida; b Morsani College of Medicine, University of South Florida, Tampa, Florida; c Heart and Vascular Center, Dartmouth-Hitchcock Medical Center, Lebanon, New Hampshire; d Baptist Heart and Vascular Institute, Central Baptist Hospital, Lexington, Kentucky; e Medical Affairs, Gentuity LLC, Sudbury, Massachusetts; f Department of Radiology, University of Massachusetts Chan Medical School, Worcester, Massachusetts; g Advanced Development, Spryte Medical LLC, Bedford, Massachusetts

**Keywords:** image-guided percutaneous coronary intervention, intravascular imaging, optical coherence tomography

## Abstract

**Background:**

Optical coherence tomography (OCT) has emerged as an essential tool in coronary atherosclerosis research and has shown clinical value in optimizing percutaneous coronary intervention. Its capability to identify coronary plaque pathology and accurately detect intervention results, often overlooked by angiography, serves as a guide in managing patients with acute coronary syndromes, myocardial infarction due to nonobstructing coronary artery disease, calcified arteries, and in-stent restenosis, thus contributing to improved clinical outcomes. However, the current technology of intracoronary imaging catheters has a size approaching 3F, limiting its adoption preintervention. Furthermore, the image field of view of current OCT technologies cannot consistently offer complete visualization of coronary arteries ≥5 mm.

**Methods:**

In this multicenter, single-arm study, we evaluated the efficacy and safety of a novel imaging catheter and system called high-frequency optical coherence tomography (HF-OCT). This system features a reduced-size, rapid-exchange imaging catheter with a diameter of 1.8F. HF-OCT captures 100 mm long segments of coronary arteries in just 1 second. In addition, HF-OCT provides an expanded field of view greater than 14 mm in diameter, enabling complete imaging of large coronary arteries.

**Results:**

After conducting 143 imaging acquisitions in 81 unique coronary arteries across 75 patients at 3 institutions, we obtained an average clear image length of 68.8 ± 18.8 mm. Coronary arteries of varying sizes, including cases with severe stenosis, were evaluated. Comparing preintervention HF-OCT acquisitions—taken prior to any arterial manipulation—to postintervention acquisitions, no significant difference in image quality was observed (*t* test, *P* = .901).

**Conclusions:**

The results of this study illustrate that a lower HF-OCT catheter profile, larger field of view, and faster pullback capabilities provide reliable imaging of coronary arteries in an all-comers, multicenter population.

## Introduction

Intracoronary optical coherence tomography (OCT) is a well-established imaging modality utilized to support percutaneous coronary interventions (PCI). Starting in 2009, the second-generation frequency-domain OCT, utilizing fast, sweep-source lasers for rapid 2-second acquisitions, has facilitated widespread adoption for coronary use.^1^, ^2^, ^3^ Utilizing a low-power near-infrared laser, OCT generates images at an axial resolution of approximately 10 to 15 μm, significantly higher than x-ray imaging modalities and intravascular ultrasound.^4^ Offering a detailed quantitative assessment of coronary pathology not available to x-ray angiography techniques, it enables accurate device sizing and placement location,^5^, ^6^, ^7^ that can result in improved clinical outcomes.^8^ In addition, it has become an indispensable tool in coronary atherosclerosis research.^9^ It is estimated that over 100,000 coronary OCT procedures per year have been performed worldwide since 2017.^10^ Current technology adoption for preintervention assessment of disease state, however, is limited by a catheter size >2.6F. In severely narrowed arteries, preintervention imaging cannot be reliably and consistently performed,^11^ as the imaging catheter fully obstructs antegrade flow beyond the stenosis, and the lesion and distal vessel cannot be cleared of blood and therefore cannot be visualized. Initial lesion preparation with an angioplasty balloon prior to imaging is often required in these cases; however, manipulation of the artery can limit the ability to accurately assess the lesion characteristics, identify acute coronary syndrome (ACS) culprit lesions, or subtype ACS presentations such as plaque rupture, plaque erosion, and eruptive calcified nodules. Furthermore, the current OCT technology cannot offer a complete visualization of large coronary arteries, due to a limited field of view.

Recently, a novel OCT modality has been proposed, named high-frequency OCT (HF-OCT)^12^, ^13^, ^14^ using a reduced-size catheter with a maximum diameter of 1.8F.^15^ In addition to a miniaturized catheter, HF-OCT acquires coronary images at a pullback speed of up to 100 mm/s, enabling imaging of a 100 mm long coronary segment in 1 second, with an image field of view (diameter) larger than 14 mm. In initial single-center studies, HF-OCT was demonstrated to capture high-quality image data of stenosed coronary arteries with a minimum lumen area (MLA) as small as 0.35 mm^2^ without the use of balloon angioplasty.^15^ The faster HF-OCT pullback speed was shown to be able to capture long segments of coronary arteries using a reduced amount of contrast (ie, as low as 5 mL per acquisition) without affecting data quality.^16^ In this study, we report the first multicenter experience to evaluate the safety and efficacy of HF-OCT for the imaging of coronary arteries in a series of 75 patients.

## Methods

### Study population

This single-arm, multicenter study included 3 medical centers (Tampa General Hospital, Tampa, FL; Dartmouth-Hitchcock Medical Center, Lebanon, NH; and Baptist Health, Lexington, KY) and 10 different operators, and was conducted from February 2021 to January 2023. All patients provided written informed consent and enrolled under a WCG IRB-approved study protocol. The study was registered in ClinicalTrials.gov (NCT04533503). Patients were all-comers and were included in the study if they were adults (aged >18 years), presenting for cardiac catheterization for stable coronary artery disease (CAD) or ACS, and were considered candidates for PCI. Study exclusion criteria included the following: bacteremia or sepsis, major coagulation system abnormalities, severe hemodynamic instability or shock, acute renal failure, disqualified for coronary artery bypass graft surgery, disqualified for PCI, patients who were enrolled in another study to evaluate an investigational device or medication. Lesion-specific exclusion criteria included the following: total occlusion, coronary artery spasm, large thrombus burden, and any target vessel that has undergone a bypass procedure. No aorta-ostial lesions were included in this study.

### HF-OCT imaging system and catheter

**Figure 1:**
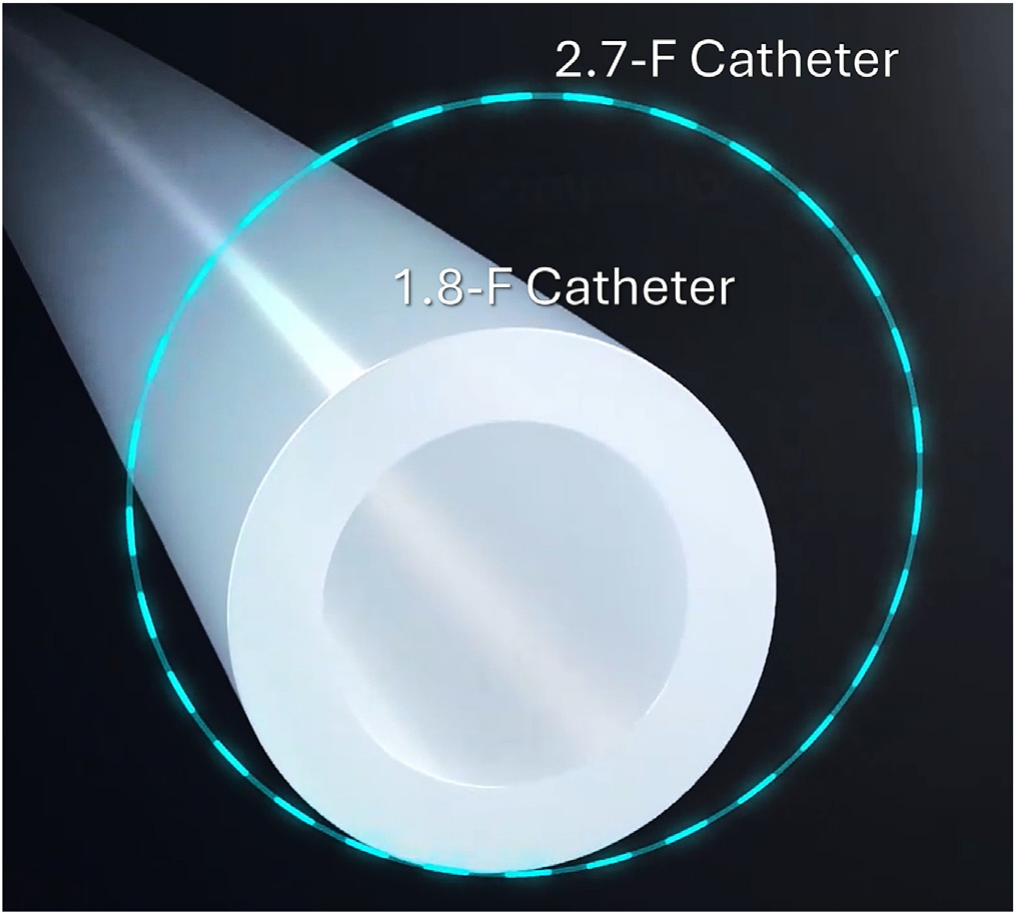
**The image shows the high-frequency optical coherence tomography catheter size in comparison with traditional intravascular imaging catheters.** The high-frequency optical coherence tomography imaging catheter has an outer diameter of 1.8F, representing a reduction in cross-sectional area >50% with respect to conventional optical coherence tomography imaging catheters that are 2.7F in size or larger.

The imaging catheter (Vis-Rx, Gentuity LLC) is a 1.8F, rapid-exchange coronary device with an insertable length of 165 cm.^15^ Vis-Rx miniaturization solutions enabled a cross-sectional area approximately 50% smaller than currently available OCT technologies (Figure 1).^17^ The HF-OCT console uses a rapid swept-source near-infrared laser with a central wavelength of approximately 1310 nm, collecting 200,000 A-scan lines per second. The catheter and system collect images at a rate of 250 frames per second, acquiring HF-OCT images from a coronary segment up to 100 mm long in 1 second, with a pullback speed approximately two and a half times faster than existing OCT technologies.^18^ HF-OCT axial resolution approaches 10 μm in tissue (refractive index n = 1.45), and the lateral resolution of the Vis-Rx catheter optics is approximately 35 μm at focus. The system's scan range in contrast is greater than 14 mm in diameter, allowing complete imaging of large coronaries (eg, left main [LM]). Additionally, the console software provides rapid, fully automatic, artificial intelligence–based quantitative measurements of the arterial lumen while allowing users to review and manually adjust measurements and tracings obtained automatically.

### HF-OCT image acquisition

Procedures were conducted via either a radial or femoral approach through a 6F coronary guiding catheter. HF-OCT was used at various stages of the intervention, including before and after stent placement, at the operator's discretion. Similarly, preintervention imaging was acquired with or without vessel predilation. As is routine for OCT imaging, data were collected during a brief contrast injection, utilizing either a standard angiographic 10-mL hand injection or an automated injector. When a power injector was used, it was set to inject contrast media at 4 mL/s, with a total volume ranging between 10 mL and 14 mL. Imaging was repeated as necessary to ensure the acquisition of all relevant information needed for the intervention.

### Vessel assessment and clear image length analysis

High-frequency OCT pullbacks were analyzed by an independent core laboratory (University Hospitals Cleveland Medical Center) to determine clear image length (CIL). Additional analyses were performed by author D.L.Q. Similar to previous studies, a cross-section is defined as “clear” when the vessel wall is visible along at least an arc of 270° around the center of the lumen.^19^ CIL (mm) was calculated as the length of the pullback segment containing “clear” images. CIL was also quantified as a percentage, being defined as the CIL (mm) compared to a given length of the pullback, excluding the guide catheter length. The MLA was identified and quantified for each data set, as well as the proximal and distal vessel size. Pre-PCI pullbacks (prior to any vessel manipulation) were further evaluated by assessing the CIL distal to the MLA.

### Statistical analysis

Unless indicated otherwise, data are presented as means and SD. Data association was evaluated with a *t* test. A 2-tailed *P* <.05 was considered statistically significant.

## Results

**Figure 2:**
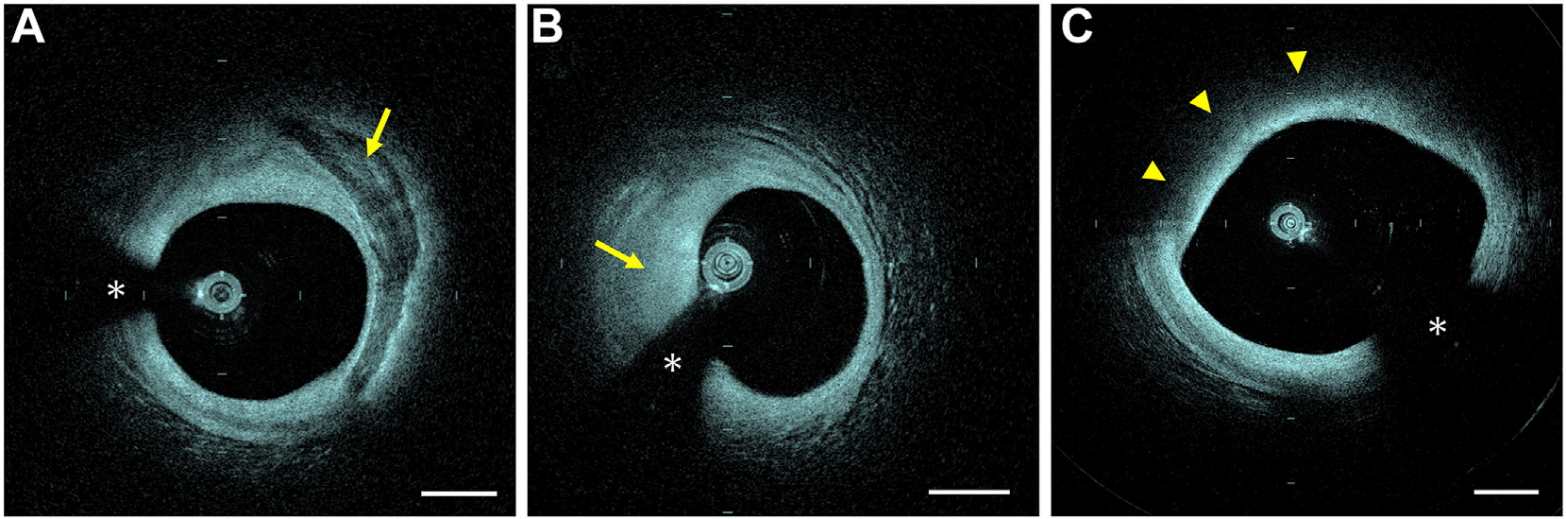
**The images show examples of high-frequency optical coherence tomography imaging of coronary atherosclerosis.** (A) The high-frequency optical coherence tomography cross-sectional image shows a calcific plaque (arrow). (B) The image shows a fibrotic plaque (arrow), and (C) shows a large eccentric lipid pool (arrowheads). The asterisk indicates the guide wire shadowing artifact. Scale bars are equal to 1 mm.

A total of 75 patients underwent HF-OCT imaging, resulting in the successful collection of 143 HF-OCT data sets in 81 unique target arteries. HF-OCT was performed using a standard angiographic 10 mL hand injection in 65.7% of the acquisitions (n = 94) and using an automated injector in 34.3% of the acquisitions (n = 49). In some instances, data set acquisitions were repeated (n = 28) due to procedural errors, such as insufficient contrast injections, guide catheter disengagement, inaccurate imaging catheter placement, and the need to replace the imaging catheter. Patient characteristics are presented in Table 1, and examples of HF-OCT imaging are illustrated in Figure 2. The left anterior descending (LAD) artery and its branches were the most frequent target arteries (n = 42, 51.8%), followed by the right coronary artery (RCA, n = 23, 28.4%), the left circumflex (LCx) artery and its branches (n = 14, 17.3%), and the ramus intermedius (n = 2, 2.5%). Imaging of the LM coronary artery, or a portion of it, was obtained in 74 acquisitions initiated from the LAD or the LCx artery. The sizes of the coronary arteries undergoing HF-OCT imaging are presented in Table 2. We observed an average CIL of 68.8 ± 18.8 mm (n = 143), with a median value of 68.4 mm. Preintervention HF-OCT pullbacks, prior to any vessel manipulation, including balloon predilation, showed a CIL of 68.6 ± 17.3 mm (n = 50). Postintervention CIL was 69.0 ± 19.5 mm (n = 93). No significant difference was found (ie, CIL values) between preintervention and postintervention acquisitions (*t* test, *P* = .901). Additional quantitative CIL analyses are presented in Table 2. No procedural complications related to the use of HF-OCT were observed, including coronary artery dissection, coronary artery perforation, or abrupt vessel closure. Additionally, there were no serious postprocedural adverse events, such as myocardial infarction, urgent percutaneous or surgical revascularization, sustained ventricular arrhythmias, or death.

**Table 1:** Patient demographic.

Patient demographics	N = 75
Age, y	67.2 ± 10.1
Male sex	54 (72.0%)
Body mass index, kg/m^2^	30.7 ± 6.3
Prior PCI	37 (49.3%)
Diabetes mellitus	31 (41.3%)
Hypertension	68 (90.7%)
Statin use	57 (76.0%)
Smoking status	
Current	11 (14.7%)
Former	30 (40.0%)
Never	34 (45.3%)
Patient presentation	
Silent ischemia	7 (9.3%)
Stable angina	20 (26.7%)
Unstable angina	22 (29.3%)
NSTEMI	11 (14.7%)
STEMI	3 (4.0%)
Others	12 (16.0%)

NSTEMI, non-ST-segment elevation myocardial infarction; PCI, percutaneous coronary intervention; STEMI, acute ST-segment elevation myocardial infarction.

**Table 2:** HF-OCT quantitative analysis summary.

Quantitative HF-OCT analysis	N = 143
Proximal reference vessel areas, mm^2^	Range, 3.2-27.6
Distal reference vessel areas, mm^2^	Range, 1.2-14.6
MLA, mm^2^	Range, 0.4-9.5

CIL quantitative analysis	

CIL, all data sets, mm	68.8 ± 18.8 (n = 143)
CIL, pre-intervention data sets, mm	68.6 ± 17.3 (n = 50)
CIL, post-intervention data sets, mm	69.0 ± 19.5 (n = 93)
CIL, pre-intervention data sets from arteries with a proximal reference diameter ≥3.5 mm, mm	67.5 ± 17.4 (n = 30)
CIL, pre-intervention data sets including severe stenosis with MLA <0.63 mm^2^, mm	60.7 ± 6.8 (n = 5)
CIL, pre-intervention data set subsegments distal to the MLA location	>90% in 88% of the acquisitions (n = 50)

Values are mean ± SD unless otherwise indicated.

CIL, clear image length; HF-OCT, high-frequency optical coherence tomography; MLA, minimum lumen area.

### Examples of HF-OCT imaging

**Figure 3:**
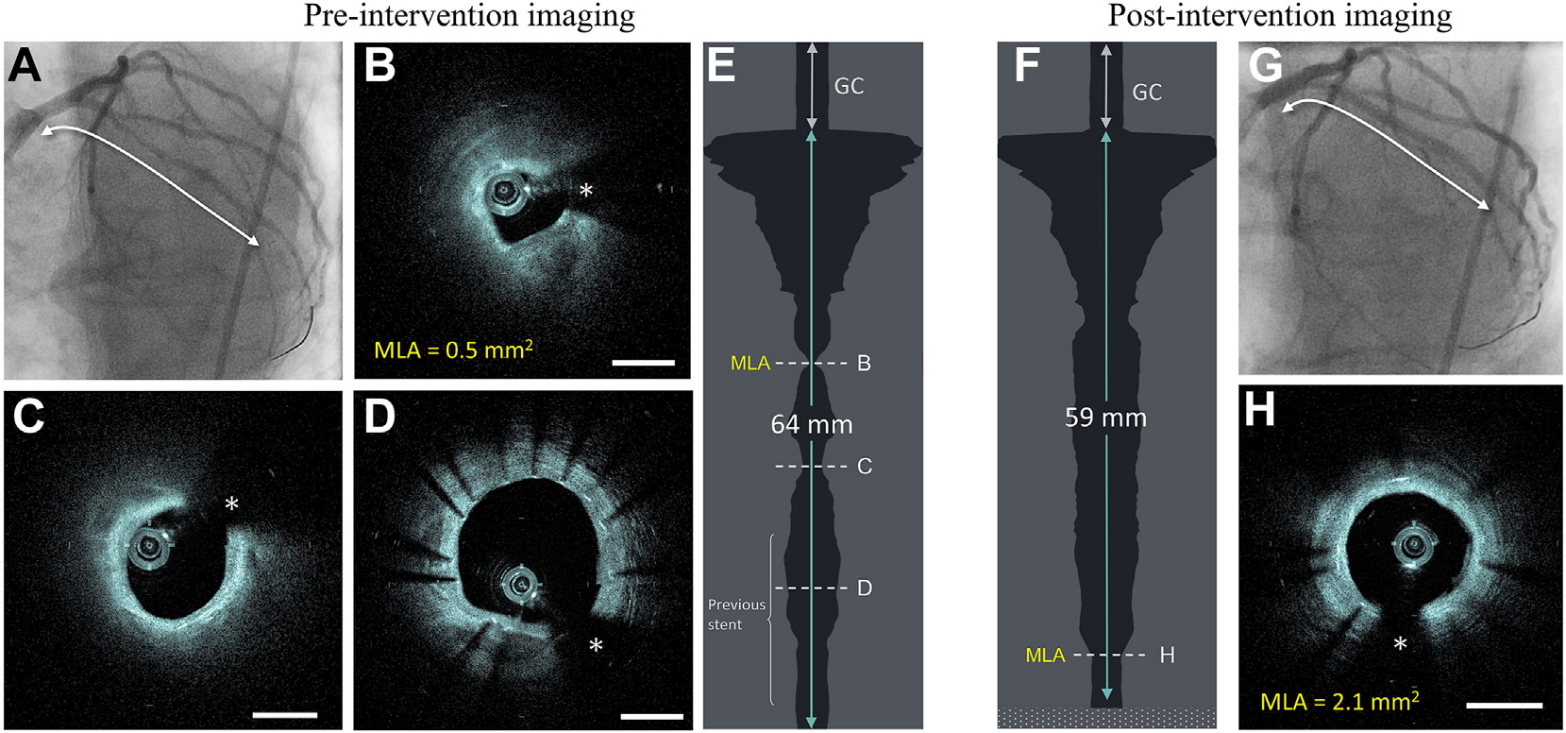
**(A) The image showsx-ray angiography of a previously stented left circumflex artery preintervention.** (B) The high-frequency optical coherence tomography (HF-OCT) cross-shows an MLA equal to 0.5 mm^2^ located proximally to the stented region. (C) The image shows a second, more distal, luminal narrowing. (D) HF-OCT imaging of the previous stent, showing a thin layer of neointimal tissue. (E) Despite the tight lumen area, HF-OCT images free of blood were obtained, including the vessel segment distal to the MLA. (F) The image shows the lumen profile image postintervention. (G) The x-ray image shows postintervention angiographic results. (H) The HF-OCT image shows the MLA site postintervention. The asterisk indicates the guide wire shadowing artifact. Scale bars are equal to 1 mm. GC, guide catheter; MLA, minimum lumen area.

**Figure 4:**
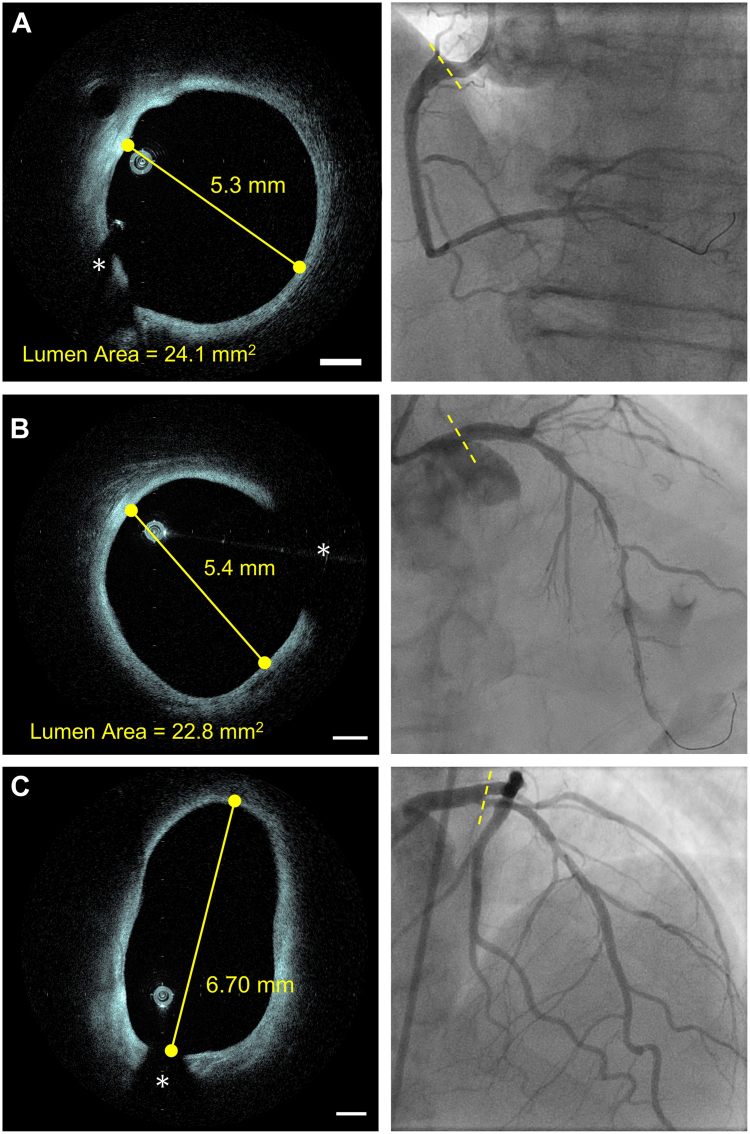
**Examples of high-frequency optical coherence tomography (HF-OCT) imaging in arteries with a lumen diameter of 5 mm or larger.** (A) The image shows an example of HF-OCT ostial right coronary artery imaging. The cross-section shows a lumen area of 24.1 mm^2^. (B) The image shows HF-OCT imaging of a left main coronary artery. (C) The image shows HF-OCT imaging of the left main bifurcation. Despite the large lumen area and diameters, HF-OCT acquired a complete view of the arterial wall. The asterisk (∗) indicates the guide wire shadowing artifact. The scale bars are equal to 1 mm in all images.

The Central Illustration presents an example of preintervention imaging in a tightly stenosed LAD artery, estimated to be approximately 90% on angiography. HF-OCT imaging was performed pre-intervention without the use of balloon angioplasty or vessel preparation. An MLA of 0.68 mm^2^ was observed, and HF-OCT captured a 90 mm long segment with images clear of blood. Blood-free images were obtained both at the level of the MLA and distal to it, encompassing a distal segment with an arterial diameter of less than 2 mm, providing a comprehensive assessment of the disease state. A second example is presented in Figure 3, where a patient underwent HF-OCT imaging to assess the disease state of a previously stented LCx coronary artery with angiographically visible stenoses. Intravascular imaging revealed high-grade stenosis with an MLA of 0.5 mm^2^ located proximal to the previously stented segment, with no evidence of in-stent restenosis. Despite the tight stenosis, images distal to the lesion were captured free of blood-shadowing artifacts. Figure 4A, B illustrates examples of ostial RCA imaging and LM imaging. Due to its extended field of view, HF-OCT demonstrated the capability to image arteries with large lumens, measuring 24.1 mm^2^ at the RCA ostium and 22.8 mm^2^ in the LM artery, without cropping any portion of the vessel. This was also achieved in cases where the imaging catheter was seated in an eccentric position within the arterial lumen. In Figure 4C, an example of LM bifurcation imaging with HF-OCT is depicted, fully capturing the ostium and the lumen of both the LCx and the LAD artery.

**Central Illustration:**
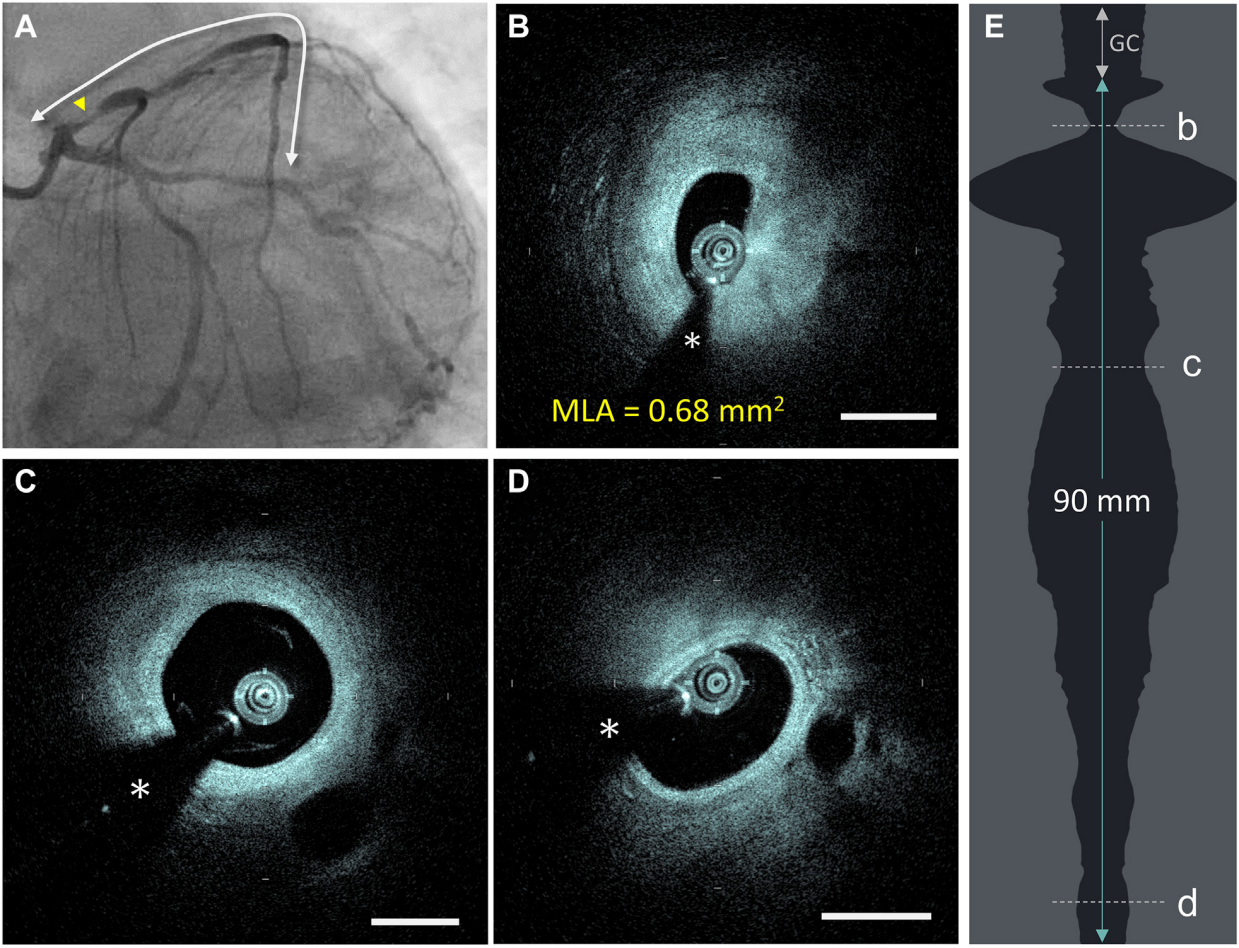
The images show an example of high-frequency optical coherence tomography (HF-OCT) preintervention imaging in a severely stenosed coronary artery. (A) X-ray angiography shows a 90% lesion at the level of the proximal left anterior descending artery. (B) The HF-OCT cross-sectional image shows the MLA site, with a lumen size of 0.68 mm^2^. (C, D) the images show complete blood clearance proximal and distal to the MLA location, offering a complete assessment of the artery disease. (E) The image shows the HF-OCT lumen profile. A 90 mm long clear segment of the artery was obtained in 1 second by injecting contrast using a 10-cc manual syringe injection. Scale bars on all images are equal to 1 mm. The asterisk (∗) indicates the guide wire shadowing artifact. GC, guide catheter; MLA, minimum lumen area.

## Discussion

This study evaluated the efficacy and safety of HF-OCT in patients undergoing PCI across 3 different centers. Data acquired by multiple different operators demonstrated safe and consistently clear intracoronary imaging across various patient presentations and CAD, including high-grade stenoses and diffuse lesions, before and after interventions. Advancements in the miniaturization of the HF-OCT imaging catheter, combined with an increased pullback speed (ie, 100 mm/s) enabled operators to achieve imaging of long segments of coronary arteries in just 1 second using brief manual or automated contrast injections. Consistent with previous data,^15^ in this study, HF-OCT could obtain clear blood-free images in tightly stenosed arteries, both proximal and distal to the lesion. The extended field of view allowed for imaging of large arteries, including the LM trunk and proximal vessel segments of the RCA. Furthermore, the utilization of a miniaturized imaging catheter holds the potential to address scenarios where existing OCT and intravascular ultrasound technologies face challenges in successful navigation and delivery to the region of interest. This is particularly relevant in complex cases involving high-grade stenoses, which limit the ability to perform preintervention intravascular imaging. Furthermore, the faster pullback speed could facilitate imaging acquisitions using saline injections instead of contrast; however, this was not investigated in the current study.

Previous studies have demonstrated that intravascular imaging significantly influences operator decision-making, especially in the preintervention phase.^20^ By precisely measuring lumen and vessel (ie, external elastic lamina) diameters, routine use of HF-OCT preintervention can help provide optimal guidance for stent sizing and accurately define landing reference locations. Furthermore, routine HF-OCT preintervention imaging can guide the safe use of direct stenting or the implementation of an optimized preparation strategy,^21^ and allow for the application of computational methods for deriving fractional flow reserve (FFR) measurements.^22^ As a matter of fact, in the case of soft lipid plaques, OCT can support direct stenting without the necessity for predilation,^23^ potentially reducing the risk of “no-reflow” phenomenon. Similarly, it can help avoid placing reference segments at the level of large lipid plaques, and, if lipid presence in the reference segment is unavoidable, imaging provides the option of intentionally covering the entire lipid-rich plaque with a stent instead of terminating the stent in the middle of a lipidic region. For calcified lesions, often underestimated by angiography, it enables quantification of both plaque thickness and extent, informing the use of plaque modification techniques such as atherectomy, intravascular lithotripsy, cutting, or noncompliant balloons to achieve optimal stent expansion.^24^^,^^25^ Similarly, preintervention imaging aids in detecting calcified nodules before stent placement, a known predictor of stent failure.^26^ When assessing severe in-stent restenosis, HF-OCT evaluation without the use of balloon angioplasty might offer a detailed diagnosis without committing to an endovascular intervention strategy.^27^ Compared with angiography guidance, intravascular imaging guidance was shown to enhance both the safety and effectiveness of PCI.^28^ The application of OCT in complex bifurcation treatments has also shown superior clinical outcomes, with lower 2-year major adverse cardiac events compared to angiography-guided procedures.^29^^,^^30^ However, despite data showing that the use of intravascular imaging has been proven to improve PCI results,^8^^,^^31^, ^32^, ^33^, ^34^ the global utilization of OCT remains low. When using traditional intravascular imaging technologies, difficulties arise when attempting to image large arteries or long segments.^19^ Moreover, in severely stenosed arteries, a large imaging catheter can be obstructive, which can lead to ischemia and poor imaging. As such, users often avoid the use of OCT in these situations. The data presented here show that the use of HF-OCT can provide complete vessel imaging using rapid acquisition through a miniaturized imaging catheter, facilitating consistent and frequent use of intravascular imaging in both treated and untreated arteries with a broad range of size and degree of stenosis.

### Study limitations

The sample size evaluated in this study was limited to 75 patients and 81 unique coronary arteries. Although an all-comers patient population was investigated and successful imaging with long CIL was observed, future studies will need to evaluate the performances of HF-OCT in a larger patient population.

## Conclusion

Utilizing a low-profile imaging catheter and a console with an improved imaging speed and a larger image field of view, HF-OCT introduces novel intravascular imaging capabilities. This technology has demonstrated the ability to consistently acquire high-quality images in both tightly stenosed arteries and large lumens, facilitating a consistent and comprehensive assessment of CAD. Coupled with a faster imaging speed, the reduced-size HF-OCT catheter can capture coronary segments up to 100 mm long in just 1 second, effectively addressing some of the limitations associated with existing OCT devices. These advancements position HF-OCT as a valuable tool to support routine intravascular imaging in clinical settings.
